# Phosphoinositides: Roles in the Development of Microglial-Mediated Neuroinflammation and Neurodegeneration

**DOI:** 10.3389/fncel.2021.652593

**Published:** 2021-03-26

**Authors:** Thomas Ernest James Phillips, Emily Maguire

**Affiliations:** UK Dementia Research Institute at Cardiff University, Cardiff, United Kingdom

**Keywords:** microglia, neurodegeneration, neuroinflammation, phosphoinositols, Alzheimer’s disease, Parkinson’s disease, phagocytosis, chemotaxis

## Abstract

Microglia are increasingly recognized as vital players in the pathology of a variety of neurodegenerative conditions including Alzheimer’s (AD) and Parkinson’s (PD) disease. While microglia have a protective role in the brain, their dysfunction can lead to neuroinflammation and contributes to disease progression. Also, a growing body of literature highlights the seven phosphoinositides, or PIPs, as key players in the regulation of microglial-mediated neuroinflammation. These small signaling lipids are phosphorylated derivates of phosphatidylinositol, are enriched in the brain, and have well-established roles in both homeostasis and disease.Disrupted PIP levels and signaling has been detected in a variety of dementias. Moreover, many known AD disease modifiers identified *via* genetic studies are expressed in microglia and are involved in phospholipid metabolism. One of these, the enzyme PLCγ2 that hydrolyzes the PIP species PI(4,5)P_2_, displays altered expression in AD and PD and is currently being investigated as a potential therapeutic target.Perhaps unsurprisingly, neurodegenerative conditions exhibiting PIP dyshomeostasis also tend to show alterations in aspects of microglial function regulated by these lipids. In particular, phosphoinositides regulate the activities of proteins and enzymes required for endocytosis, toll-like receptor signaling, purinergic signaling, chemotaxis, and migration, all of which are affected in a variety of neurodegenerative conditions. These functions are crucial to allow microglia to adequately survey the brain and respond appropriately to invading pathogens and other abnormalities, including misfolded proteins. AD and PD therapies are being developed to target many of the above pathways, and although not yet investigated, simultaneous PIP manipulation might enhance the beneficial effects observed. Currently, only limited therapeutics are available for dementia, and although these show some benefits for symptom severity and progression, they are far from curative. Given the importance of microglia and PIPs in dementia development, this review summarizes current research and asks whether we can exploit this information to design more targeted, or perhaps combined, dementia therapeutics. More work is needed to fully characterize the pathways discussed in this review, but given the strength of the current literature, insights in this area could be invaluable for the future of neurodegenerative disease research.

## Introduction

Fifty million people worldwide currently present with neurodegenerative conditions, with 60–70% of these suffering from Alzheimer’s disease (AD) (World Alzheimer Report, 2015). Microglia, tissue-specific macrophages that reside in the central nervous system (CNS), are becoming increasingly recognized as important in the development of numerous dementia pathologies (Bachiller et al., 2018). Unlike most macrophages, microglial precursors emerge from the embryonic yolk sac and migrate into the CNS during the first trimester of development before their final maturation (Kierdorf and Prinz, 2013).

During development, microglia have key roles in shaping neuronal networks and modulating both the number of synapses and the strength of synaptic transmission (Colonna and Butovsky, 2017). Following CNS injury, microglial phagocytosis of various substrates including microbes, dead cells, and protein aggregates helps maintain healthy brain homeostasis (Gabandé-Rodríguez et al., 2020). Furthermore, these cells secrete messenger molecules such as cytokines, chemokines, and neurotrophic factors (Lee et al., 2002). Cytokine secretion regulates inflammatory responses, whilst chemokines initiate chemotaxis and migration, stimulating microglia and other immune cells to become activated and migrate to the site of injury (Lee et al., 2002). Both phagocytosis and cytokine/chemokine secretion can be triggered *via* activation of microglial toll-like receptors (TLRs), which promote inflammation in response to activation by pathogen-associated molecular patterns (PAMPs) and other stimuli (Fiebich et al., 2018). Microglia are also key modulators of purinergic signaling, with activation of these pathways influencing both inflammation and phagocytosis (Calovi et al., 2019).

Phosphoinositides (PIPs), in brief, are acidic membrane lipids derived from phosphatidylinositol. These lipids, known to support key cellular functions in the brain, are increasingly recognized as important in neurodegenerative processes and microglial function (Raghu et al., 2019).

This review aims to summarize the role of microglia in a variety of neurodegenerative conditions, as well as known phosphoinositide disturbances within these conditions. This will be followed by discussions on how alterations in phosphoinositides and their regulatory enzymes could affect specific microglial functions and thereby contribute to disease progression. Finally, for each microglial function discussed, we will explore how phosphoinositide-modifying therapies could potentially be used to ameliorate disease phenotypes.

## Roles of Microglia in Dementia

### Alzheimer’s Disease

AD, first characterized by Alois Alzheimer in 1907 (Alzheimer et al., 1995), presents with widespread brain atrophy, amyloid plaques (large extracellular deposits of amyloid-beta (Aβ) protein aggregates), neurofibrillary tangles (consisting of phosphorylated Tau), neuronal and synapse loss, and dystrophic neurites (Lane et al., 2018). Clinically, these pathologies result in memory loss, language difficulties, executive dysfunction, psychiatric symptoms, and behavioral disturbances, along with general difficulties managing activities of daily living (Burns and Iliffe, 2009). AD can be either familial (<0.5% of cases) or sporadic. Familial cases arise following mutations in the genes encoding either presenilin 1 (PSEN1), presenilin 2 (PSEN2), or amyloid precursor protein (APP; Bateman et al., 2011).

Within AD, we know that 58–79% of sporadic cases are linked to the patient’s genes (Gatz et al., 2006). Genetics studies strongly suggest microglia as a leading driver of AD pathology, with many of the implicated genes either largely or solely expressed in microglia (McQuade and Blurton-Jones, 2019). The importance of microglia is supported by observations of proliferation and activation of microglia around amyloid plaques (Hickman et al., 2008). Whether this microglial response reduces disease progression, enhances AD pathology, or both is currently the subject of much debate. AD microglia elicit a range of functional changes including increased cytokine/chemokine production and inflammasome activation, increased synapse engulfment, and phagocytosis of injured but functional neurons (McQuade and Blurton-Jones, 2019). The role of microglia in Aβ clearance within AD is also unclear. Whilst microglial phagocytosis of Aβ appears crucial in clearing plaques (Simard et al., 2006), other studies have shown that pharmacological depletion of microglia prevents plaque formation in the first place (Sosna et al., 2018).

Nitric oxide (NO) curative therapy currently exists for the treatment of AD (Weller and Budson, 2018). This may be due to a large research focus in the past on the “amyloid hypothesis” which postulates that all AD pathologies arise due to Aβ accumulation and plaque formation, and consequently that the best way to treat the disease is to target Aβ directly (Oxford et al., 2020).

### Parkinson’s Disease

Parkinson’s disease (PD), first described by James Parkinson in 1817 (Parkinson, 2002), is the second most common neurodegenerative disease, affecting about 6.1 million people worldwide (2018). PD patients experience rigidity, bradykinesia, resting tremors, and postural instability (Gopalakrishna and Alexander, 2015), with 30% also experiencing dementia (Hanagasi et al., 2017).

Symptoms arise following the degeneration of dopaminergic neurons in the substantia nigra, which produce the neurotransmitter dopamine. Neuronal loss occurs following the formation of intraneuronal “Lewy bodies” which consist of aggregated bundles of misfolded α-synuclein (Lecours et al., 2018). Variants in the SNCA gene, which encodes for α-synuclein, present as the most well-established genetic risk factor for PD (Campêlo and Silva, 2017). Around 5% of PD cases are caused by mendelian gene changes (e.g., SNCA) and are therefore classed as familial. The remainder of PD cases arises following a complex interplay of aging, genetic susceptibility, and environmental factors (Pang et al., 2019).

Within the PD brain, microglia are thought to lose beneficial, whilst gaining detrimental, functions (Lecours et al., 2018). Positron emission tomography (PET) studies in patients, as well as work by McGeer and colleagues on post-mortem tissue, demonstrate increased microglial activation in PD brains (McGeer et al., 1988; Ouchi et al., 2005; Gerhard et al., 2006). Moreover, increased inflammatory cytokines, released by microglia, are observed within the brains and cerebrospinal fluid (CSF) of PD patients (Vawter et al., 1996; Nagatsu et al., 2000). CSF from PD patients is toxic to dopaminergic neurons; in part due to the aforementioned high inflammatory cytokine concentration (Nagatsu and Sawada, 2005). Furthermore, and similarly to AD, PD microglia appear to phagocytose injured but functional neurons, thereby exacerbating neurodegeneration (Brown and Neher, 2012). These microglial phenotypes seem to occur following exposure to aggregated α-synuclein (Zhang et al., 2005).

3, 4-dihydroxy-L-phenylalanine, the precursor to dopamine, acts as the “gold standard” PD treatment. Nevertheless, while this drug ameliorates many PD-associated motor defects, long-term use often results in debilitating dyskinesia and other motor fluctuations (Lane, 2019).

### Huntington’s Disease

Microglia have also been implicated in the pathology of Huntington’s disease (HD; Yang H.-M. et al., 2017). HD is an autosomal dominant trinucleotide repeat disorder caused by an expansion in the Huntington protein (HTT; MacDonald et al., 1993). More than 40 repeats result in disease, characterized primarily by dysfunction and death of neurons within the striatum of the brain. For patients, this results in progressive motor, cognitive, and psychiatric disturbances (Bates et al., 2015).

PET studies on human HD post-mortem brains have demonstrated increased activation of microglia in HD compared with controls (Pavese et al., 2006; Politis et al., 2012), with this activation occurring up to 15 years before the predicted age of onset (Tai et al., 2007). The degree of microglial activation appears to correlate positively with the degree of cell death within a given brain region, as well as symptom severity (Sapp et al., 2001). Moreover, mutated Huntington protein (mHTT) expression appears to impact microglial function directly (Yang H.-M. et al., 2017). Effects include increased cytokine production and transcriptional dysregulation (Crotti et al., 2014; Träger et al., 2014; Miller et al., 2016). There are currently no curative therapies available for HD (McColgan and Tabrizi, 2018).

### Amyotrophic Lateral Sclerosis

Amyotrophic lateral sclerosis (ALS) is a degenerative disease primarily characterized by muscle weakness and wasting, with 10–15% of patients also suffering from frontotemporal dementia (FTD). FTD results in progressive degeneration of frontal and anterior temporal lobes, with patients experiencing behavioral changes alongside impairments in executive functioning and, often, language. ALS is familial in 15% of cases, where it is caused by changes in one of more than 20 currently identified genes (Masrori and Van Damme, 2020). The most common cause (of both ALS and FTD) in North America and Europe is a hexanucleotide GGGGCC expansion in the *c9orf72* gene (Dejesus-Hernandez et al., 2011; Renton et al., 2011). The function of the c9orf72 protein is currently unknown, although it is suspected to be involved in endocytic trafficking and autophagy (Braems et al., 2020). Within cells, cytoplasmic aggregations of TDP-43 occur in 95% of ALS patients (Masrori and Van Damme, 2020). Sporadic ALS likely occurs following complex interactions between risk loci—several of which have been identified *via* genome-wide association studies (GWAS)—and the environment (Ajroud-Driss and Siddique, 2015).

As with other dementias, the role of microglia in ALS appears to be highly complex. C9orf72 knock-out mice, while showing no motor-neuron degeneration, show altered immune responses in microglia and macrophages, highlighting the importance of these myeloid cells in ALS pathogenesis (O’Rourke et al., 2016). Furthermore, another ALS risk gene, TBK1, is involved in the production of inflammatory cytokines (Ahmad et al., 2016). Finally, microglia appear to be activated in ALS patients’ brains, with this activation occurring before the onset of clinical symptoms (Geloso et al., 2017). More research must be undertaken to fully elucidate the role of microglia in ALS pathology.

### Summary

Neurodegenerative conditions pose serious health and economic costs to our society. If left unchecked, cases are expected to triple by 2050 (Prince et al., 2013). At present, treatment is limited by a lack of effective therapies for many forms of neurodegenerative disease, despite decades of research aimed at developing such therapies. It is becoming clear that microglia, the primary mediators of neuroinflammation, play important roles in the pathologies of many forms of neurodegeneration. Further research exploring the roles of microglia to target them therapeutically may well hold the key to releasing the deadlock on treatment development.

## Roles of Phosphoinositides in Dementia

### What are Phosphoinositides?

**Figure 1:**
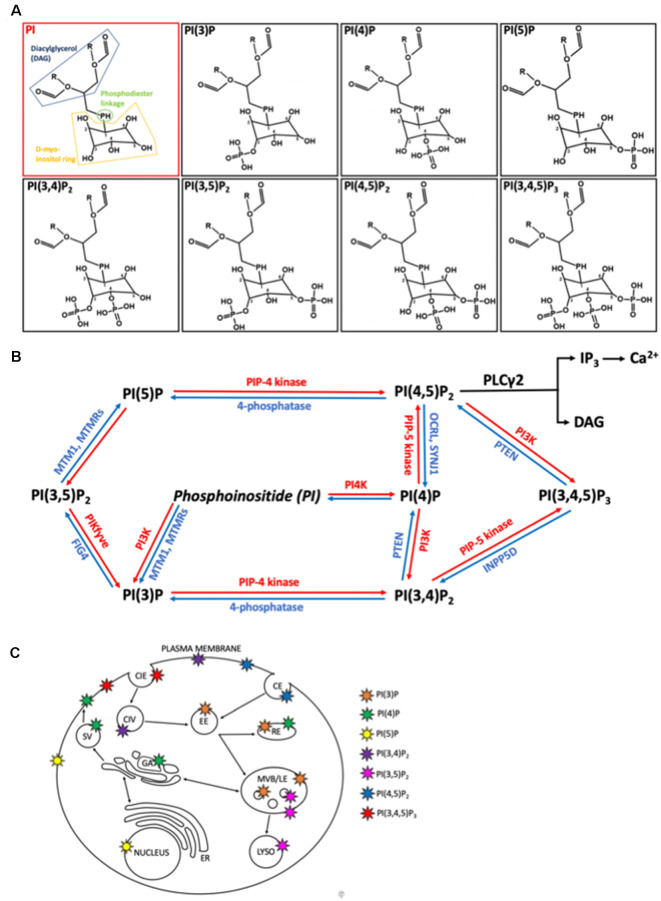
Structure, metabolism, and location of phosphatidylinositides (PIPs) within mammalian cells. **(A)** Structures of phosphatidylinositol (PI) and it is seven phosphoinositide (PIP) derivatives, generated by phosphorylation of the inositol ring at positions 3, 4 or 5. PI consists of diaglycerol (DAG, blue) bound to a D-myo-inositol ring (yellow) *via* a phosphodiester linkage (green). O, oxygen; H, hydrogen; P, phosphate; R, non-polar fatty acid tails. **(B)** Metabolic pathways regulating the interconversion of PIP species. Lipid kinases (red) phosphorylate the inositol ring at points 3, 4, or 5 to generated more phosphorylated PIPs while lipid phosphatases remove phosphate groups. MTM1, myotubularin1; MTMR, myotubularin-related protein; FIG4, Factor-Induced Gene 4; PTEN, phosphatase and tensin homolog; OCRL, inositol phosphatase 5-phosphatase; SYNJ1, synaptojanin 1; INPP5D, Src homology 2 (SH2) domain containing inositol polyphosphatase 5-phosphatase 1. **(C)** Primary locations of the different PIPs within the cell are shown by the colored stars. CIE, clathrin independent endocytosis; CIV, clathrin independent endocytic vesicle, CE, clathrin dependent endocytosis; EE, early endosome; RE, recycling endosome; SV, secretory vesicle; GA, golgi apparatus; ER, endoplasmic reticulum; MVB/LE, multi-vesicular body/late endosome; LYSO, lysosome.

Phosphoinositides are signaling lipids derived from phosphatidylinositol, which is comprised of diacylglycerol (DAG) moiety linked to a D-myo-inositol ring *via* a phosphodiester linkage. Specific kinases and phosphatases add or remove phosphate groups from the 3, 4, or 5 positions of the myo-inositol ring, generating seven PIP species. These are monophosphorylated PI(3)P, PI(4)P, and PI(5)P; bisphosphorylated PI(3,4)P_2_, PI(3,5)P_2_, and PI(4,5)P_2_; and trisphosphorylated PI(3,4,5)P_3_ (Figure 1). These lipids are enriched in the brain, with each residing on specific cellular membranes (Figure 1). In brief, PI(4)P, PI(3,4,5)P3, PI(3,4)P2, PI(5)P, and PI(4,5)P2 can be found on the plasma membrane, PI(3)P and PI(3,5)P2 are concentrated within the endocytic system, and PI(5)P is found within the nucleus. The distribution of PIPs is both dynamic and highly regulated, allowing for a rapid generation or reduction of specific species at precise locations. This function is achieved *via* tight spatial and temporal restrictions of the aforementioned PIP metabolism enzymes. Despite their low abundance, these lipids are involved in crucial cellular functions, including signal transduction, cytoskeletal reorganization, membrane dynamics, vesicular trafficking, and cell death (Phan et al., 2019). Known functions of the different PIP species in the brain are summarized in Table 1. All species appear to be involved in endocytic trafficking events (e.g., autophagy and phagocytosis), whilst others exhibit key roles in chemotaxis (PI(4,5)P_2_ and PI(3,4,5)P_3_), and synaptic function (PI(3)P, PI(4,5)P_2_, PI(3,5)P_2_, PI(3,4,5)P_3_).

**Table 1:** Known functions of phosphoinositide (PIP) species within the brain and roles in neurodegeneration.

PIP species	Known functions in the brain	Roles in neurodegenerative disease	References
PI(3)P	Key regulator of endocytic trafficking, fusion, and autophagy. Regulates GABAergic neurotransmission at inhibitory post-synapses.	Inhibiting PIP-4 kinase (phosphorylates PI(3)P) reduces mHTT and rescues neurodegeneration in HD drosophila. Excess PI3K (generates PI(3)P) activity in AD, reduced activity in PD.	Heras-Sandoval et al. (2014), Al-Ramahi et al. (2017), Papadopoulos et al. (2017) and Raghu et al. (2019)
PI(4)P	Potential roles in myelin formation. Key role in multiple steps of phagocytosis and other uptake systems.	Pathophysiological concentrations of Aβ inhibit PI4K (generates PI(4)P) activity, both *in vitro* and *in vivo*. PI4K inhibition reduces brain pathology in Drosophila models of AD. VAPB, a causal gene for ALS, exerts deleterious effects in the brain by altering PI(4)P levels and distribution. Reduced in AD cortex.	Stokes and Hawthorne (1987), Wu et al. (2006), Levin et al. (2017), Zhang X. et al. (2017), Genevini et al. (2019) and Baba et al. (2020)
PI(5)P	Roles in AKT/mTOR signaling, autophagy, and apoptosis. Regulates chromatin function and transcription in the nucleus. Potential regulators of endosomal trafficking.	Inhibiting PIP-4 kinase [phosphorylates PI(5)P] reduces mHTT and rescues neurodegeneration in HD drosophila.	Boal et al. (2015), Bulley et al. (2015), Vicinanza et al. (2015), Al-Ramahi et al. (2017) and Jacobsen et al. (2019)
PI(3,4)P_2_	Involved in the maturation of late-stage clathrin-coated pits and fast endophilin-mediated endocytosis. Roles in actin-mediated neurite initiation and dendrite morphogenesis.	Mutations in the PI(3,4)P_2_ synthesis enzyme INPP5D increase genetic AD risk. Excess PI3K [generates PI(3,4)P2] activity in AD, reduced activity in PD.	Lambert et al. (2013), Heras-Sandoval et al. (2014), Hawkins and Stephens (2016), Jing et al. (2016), Zhang S.-X. et al. (2017) and Casamento and Boucrot (2020)
PI(4,5)P_2_	Electrical signaling at the plasma membrane (including neurons). Roles in the recycling of synaptic vesicles and synaptic plasticity. Many neurotransmitters utilize the G-protein coupled PLC mediated hydrolysis of PI(4,5)P_2_ as a key step in signal transduction. Regulates ~100 ion channels and transporters. Regulates cytoskeletal function in neurons. Key regulators of TLR and purinergic signaling. Key role in actin remodeling during chemotaxis. Key role in multiple steps of phagocytosis and other uptake systems.	A genetic variant in PLCγ2, which breaks down PI(4,5)P_2_, protects against AD. Decreased PI(4,5)P_2_ metabolism *via* PLCγ2 in PD, increased PI(4,5)P_2_ in PD substantia nigra. Overexpression of SYNJ1, which hydrolyzes PI(4,5)P_2_, acts as a risk factor for AD and appears to contribute to plaque pathology and behavioral deficits in mouse models. Mutations in SYNJ1 associated with early-onset PD.	Wallace and Claro (1993), McIntire et al. (2012), Zhu et al. (2013), Drouet and Lesage (2014), Sims et al. (2017), Miranda et al. (2018), Sekar and Taghibiglou (2018), Ben Romdhan et al. (2018), Bernier et al. (2013b), Le et al. (2014), Hille et al. (2015), Dickson and Hille (2019), Raghu et al. (2019), Xie et al. (2019) and Desale and Chinnathambi (2021)
PI(3,5)P_2_	Regulates membrane trafficking, endocytic vesicle fission/fusion, organelle pH, intracellular ion channel function. Regulates synaptic strength.	The PI(3,5)P_2_ synthesis enzyme FIG4 acts as a risk factor for ALS.	Chow et al. (2009), Dong et al. (2010) and McCartney et al. (2014)
PI(3,4,5)P_3_	Regulates neurotransmitter release. Increased PI(3,4,5)P_2_ recruits protein kinases (e.g., AKT) to the PM. Regulates purinergic signaling. Key role in actin remodeling during chemotaxis. Key role in multiple steps of phagocytosis and other uptake systems	Excess PI3K [generates PI(3,4,5)P_3_] activity in AD, reduced activity in PD. Decreased in PD substantia nigra. PTEN [degrades PI(3,4,5)P_3_] increased in PD.	Bernier et al. (2013b), Khuong et al. (2013), Heras-Sandoval et al. (2014), Maekawa et al. (2014), Schlam et al. (2015), Sekar and Taghibiglou (2018), Katan and Cockcroft (2020) and Desale and Chinnathambi (2021)

*mHTT, mutant Huntington protein; AD, Alzheimer’s disease; HD, Huntington’s disease; PD, Parkinson’s disease; ALS, amyotrophic lateral sclerosis; Aβ, amyloidβ; VAPB, VAMP associated protein B and C; AKT, Protein kinase B mammalian target of rapamycin; TLR, toll-like receptor; SYNJ1, synaptojanin 1; FIG4, Factor induced gene 4; PLC, phospholipase C*.

Perhaps unsurprisingly given their key roles in the brain, PIPs have been implicated in a wide variety of dementia’s, including AD, PD, HD, and ALS. Precise perturbances of PIP species and their suspected effects on neurodegenerative disease are later discussed and summarized in Table 1.

### Phosphoinositide Dyshomeostasis in Neurodegenerative Disease—General

Growing evidence suggests that phosphoinositide dyshomeostasis plays a role in the development of a variety of dementias. Phosphoinositides, which are relatively enriched in the brain (Hawthorne and Pickard, 1979), regulate the activity of several neurotransmitters and neuropeptides (Lo Vasco, 2018). Furthermore, phosphoinositides have key roles in Ca^2+^ signaling (Bezprozvanny, 2009) and autophagy (Palamiuc et al., 2020), which are disrupted in numerous neurodegenerative conditions.

One key protein linking phosphoinositol metabolism with several dementias is synaptojanin 1 (SYNJ1). This phosphoinositide phosphatase hydrolyzes PI(4,5)P_2_, with SYNJ1 knock-out mice showed increased PI(4,5)P_2_ in neurons alongside defects in synaptic vesicle recycling (Cremona et al., 1999). Overexpression of SYNJ1 has been seen in AD patients (Miranda et al., 2018), and downregulating SYNJ1 increases clearance of amyloid plaques while improving behavioral deficits in AD mice (McIntire et al., 2012; Zhu et al., 2013). In addition to roles in AD, mutations in SYNJ1 have been associated with early-onset atypical-Parkinson’s disease, suggesting that SYNJ1 manipulation may also prove beneficial in PD (Drouet and Lesage, 2014; Ben Romdhan et al., 2018; Xie et al., 2019).

Another subset of phosphoinositide conversion enzymes with key links to dementia is the phosphoinositide-3-kinases (PI3K). PI3K promotes downstream signaling *via* AKT and mTOR and activating these pathways plays a vital regulatory role in the development of oxidative stress, in apoptosis, and in autophagy (Chong et al., 2012). Within AD, excessive activation of downstream PI3K signaling has been suggested to be responsible for some neurodegenerative processes, whereas, in PD, under-activation of these pathways has been observed to influence pathology (Heras-Sandoval et al., 2014). Activation of the Akt/PI3K signaling pathway is crucial to the initiation of neuroinflammation by microglia in response to LPS (Cianciulli et al., 2020). Although more work needs to be done to further characterize signaling dysfunctions within these conditions, targeting these pathways using established PI3K inhibitors and activators could provide potential therapeutics in the future (Yang et al., 2016, 2019).

Together, these studies demonstrate how disruptions in phosphoinositide metabolism can be crucial to the development of neurodegenerative phenotypes. The following sections will go into more detail about phosphoinositide dyshomeostasis in specific neurodegenerative conditions. Table 2 summarizes the differing roles of PIP species in neurodegenerative conditions.

**Table 2:** The role of PIP species in different neuroinflammatory conditions.

Disease	PIP Species	Suspected roles in pathology	References
Alzheimer’s disease	PI(4)P	Key role in uptake systems including phagocytosis.	Stokes and Hawthorne (1987), Wu et al. (2006), Levin et al. (2017) and Zhang et al. (2017)
	PI(3,4)P_2_	Mutations in the PI(3,4)P_2_ synthesis enzyme INPP5D increase genetic AD risk. Excess PI3K (generates PI(3,4)P_2_) activity in AD.	Lambert et al. (2013), Hawkins and Stephens (2016) and Jing et al. (2016)
	PI(4,5)P_2_	A genetic variant in PLCγ2, which breaks down PI(4,5)P_2_, protects against AD_2_, acts as a risk factor for AD.	McIntire et al. (2012) and Sims et al. (2017)
	PI(3,4,5)P_3_	Excess PI3K (generates PI(3,4,5)P_3_) activity in AD.	Heras-Sandoval et al. (2014)
Parkinson’S disease	PI(4,5)P_2_	Reduced PLC activity and PI(4,5)P_2_ metabolism in PD cortex, perhaps following the accumulation of α-synuclein which appears to inhibit PLC enzymes. Increased PI(4,5)P_2_ in PD patient substantia nigra.	Sekar and Taghibiglou (2018)
	PI(3,4,5)P_3_	Excess PI3K (generates PI(3,4,5)P_3_) reduced activity in PD_3_) increased in PD.	Bernier et al. (2013b), Sekar and Taghibiglou (2018) and Katan and Cockcroft (2020)
Huntington’S disease	PI(3)P	Inhibiting PIP-4 kinase (phosphorylates PI(3)P) reduces mHTT and rescues neurodegeneration in HD drosophila.	Al-Ramahi et al. (2017)
	PI(5)P	Inhibiting PIP-4 kinase (phosphorylates PI(5)P) reduces mHTT and rescues neurodegeneration in HD drosophila.	Al-Ramahi et al. (2017)
Amyotrophic lateral sclerosis	PI(4)P	ALS risk gene VAPB is proposed to affect neurite extension during differentiation *via* regulation of PI(4)P distribution.	Genevini et al. (2019)
	PI(3,5)P_2_	Non-synonymous variants in the PI(3,5)P_2_ phosphatase FIG4 found in 1–2% of ALS patients. LOF leads to reduced levels of PI(3,5)P_2_ and is suspected to affect autophagy.	Chow et al. (2007, 2009) and Nguyen et al. (2019)

*Aβ, amyloid β; AD, Alzheimer’s disease; INPP5D, Src homology 2 (SH2) domain containing inositol polyphosphatase 5-phosphatase 1; PLCγ2, phospholipase C γ 2; SYNJ1, synaptojanin 1; PD, Parkinson’s disease; PTEN, phosphatase and tensin homolog; mHTT, mutated Huntington protein; HD, Huntington’s disease; ALS, amyotrophic lateral sclerosis; VAPB, vesicle-associated membrane protein-associated protein B; FIG4, Factor induced gene 4; LOF, loss of function*.

### Phosphoinositide Dyshomeostasis in Alzheimer’s Disease

Quite a large body of research outlines phosphoinositide dyshomeostasis in AD. The first study to highlight this was published in 1987 by Stokes and Hawthorne. They revealed reduced PIP4 and PI(4,5)P_2_ within the AD cortex when compared with controls (Stokes and Hawthorne, 1987). Within AD brains, studies have observed not only changes in PIP levels but also changes in the expression of regulatory enzymes (Lo Vasco, 2018).

Alterations in membrane phospholipid composition within AD following PIP dysregulation could result in changes to membrane structure and fluidity, which in turn is likely to influence the development of various characteristic AD pathologies (Zhu et al., 2015). Also, Aβ binding to the cellular prion protein (PrPC) has been demonstrated to activate mGlurR5 and phospholipase C (PLC) signaling pathways (Um et al., 2013). These pathways are both regulated by and regulate phosphoinositide levels, with PI(4,5)P_2_ being the substrate of PLC enzymes. PLC activation leads to downstream cytosolic Ca^2+^ increase, which contributes to characteristic AD memory impairment (Berridge, 2013, 2014). The above observations are supported by previous studies in familial AD cortical neurons which demonstrated a clear link between Aβ and PI(4,5)P_2_ metabolism, with Aβ addition reducing PI(4,5)P_2_ levels, perhaps *via* activation of PLC enzymes (Berman et al., 2008). This could also occur *via* Aβ-mediated inhibition of the PI(4)P synthesis enzyme PI4K, as PI(4)P often acts as a precursor for PI(4,5)P_2_ (Wu et al., 2004; Figure 1B). Moreover, evidence suggests that hyperphosphorylated tau, a key hallmark of AD, may be generated by protein kinases known to be activated by PLC enzymes (Ial and Grundke-Ial, 2005).

Finally, many known AD risk genes (e.g., Phospholipase C Gamma 2 (PLCG2), inositol polyphosphate 5-phosphatase D (INPP5D), Phospholipase D3 (PLD3), CD2-associated protein (CD2AP), Phosphatidylinositol Binding Clathrin Assembly Protein (PICALM), Sodium/potassium/calcium exchanger 4 (SLC24A4)) are involved in phospholipid metabolism (Tan et al., 2019; Sims et al., 2020), providing further evidence of the importance of these pathways in disease pathology. The AD protective R522 mutation in PLCG2, which also protects against dementia with Lewy bodies and FTD (van der Lee et al., 2019), appears to protect *via* increased PLCγ2 activity (Magno et al., 2019).

### Phosphoinositide Dyshomeostasis in Parkinson’s Disease

Several studies have highlighted the specific roles of phosphoinositide dyshomeostasis in PD pathology. In one of these studies, PD and control membranes were prepared from the post-mortem prefrontal cortex and incubated with PI(4,5)P_2_ before the addition of dopamine to activate PLC. These membranes demonstrated reduced PLC activity, characterized by decreased PI(4,5)P_2_ metabolism, within the PD samples (Wallace and Claro, 1993). A major characteristic of PD and other neurodegenerative diseases, including AD, is the accumulation of α-synuclein containing inclusions in the brain (Visanji et al., 2019). α-synuclein has been shown to preferentially localize to PI(4,5)P_2_ containing membranes, where it appears to inhibit PLC enzyme activity and subsequent Ca^2+^ release (Narayanan et al., 2005), potentially explaining the results of the earlier research by Wallace and Claro (1993).

In addition to altered PLC signaling, levels of phosphatase and tensin homolog (PTEN), another phosphoinositide phosphatase, are also altered in PD. Interestingly, in this same study, PI(3,4,5)P_3_ was found to be decreased, and PI(4,5)P_2_ increased, in substantia nigra samples from PD patient brains compared to age-matched controls (Sekar and Taghibiglou, 2018). Within PD, changing phosphoinositide levels could potentially be mediated by the aforementioned α-synuclein inhibition of PI(4,5)P_2_ degrading PLC enzymes, as well as upregulation of the PI(3,4,5)P_3_ degradation enzyme PTEN.

### Phosphoinositide Dyshomeostasis in Huntington’s Disease

Phosphoinositide dyshomeostasis has also been observed in HD. For one, HTT and mHTT can be seen to interact with a variety of PIPs at membranes (Kegel et al., 2005). Interestingly, HTT interacts primarily with PI(3,4)P_2_, PI(3,5)P_2_, and PI(3,4,5)P_3_, whilst mHTT associates more strongly with PI(3,5)P_2_ than the wild-type HTT, whilst also binding to PI(3)P, PI(4)P, PI(5)P, and PI(4,5)P_2_. Changing binding affinities affects the recruitment of the Huntington protein to specific cellular membranes where distinct PIP species are located. This is likely to affect the formation of growth factor signaling complexes within mHTT cells (Kegel et al., 2009).

Studies by Al-Ramahi et al. (2017) further highlighted the roles of PIPs in HD and even demonstrated the potential of PIP regulation as a therapeutic target. This work focused on the enzyme PIP4 kinase, which phosphorylates PI(5)P and PI(3)P (Figure 1). They found that inhibiting this enzyme reduces mHTT in both patient fibroblasts and neuronal cell models. Moreover, this same study demonstrated how inhibition of PIP4 kinase rescued mHTT-induced neurodegeneration in two Drosophila HD models. This protective effect was speculated to occur *via* increased PI(3,5)P_2_. PI(5)P, PI(3)P, and PI(3,5)P_2_ all have key roles in autophagy, known to be affected in HD (Al-Ramahi et al., 2017).

### Phosphoinositide Dyshomeostasis in Amyotrophic Lateral Sclerosis

Several studies have highlighted a potential role for phosphoinositol dyshomeostasis in the pathogenesis of ALS. Firstly, non-synonymous variants in the PIP phosphatase Factor-Induced Gene 4 (FIG4) appear in 1–2% of all ALS patients (Chow et al., 2009). FIG4 regulates PI(3,5)P_2_ homeostasis, with loss of function leading to a considerable reduction in the levels of this phospholipid (Chow et al., 2007). This has been speculated to affect autophagic function (Nguyen et al., 2019). Another ALS risk gene linked to PIP homeostasis is vesicle-associated membrane protein-associated protein B (VAPB; Genevini et al., 2019). Through its various binding partners, this crucial ER adaptor protein has roles in lipid exchange, membrane traffic, Ca^2+^ signaling, cytoskeletal organization, autophagy, mitochondrial function, and neurite extension. When mutated in ALS, VAPB aggregates, forming intracellular inclusions which greatly affect ER structure. VAPB depletion appears to disrupt neurite extension during differentiation *via* reduced PI4P, presumably following its key roles regulating PI4P distribution (Genevini et al., 2019).

### Summary

Phosphoinositides have key roles in the brain, and therefore it should come as no surprise that both their levels and distribution are affected by a wide variety of neurodegenerative diseases. In several cases, this dyshomeostasis has been directly linked to disease pathology, thereby highlighting the potential of phosphoinositide-based therapies when looking to treat these devastating and often incurable conditions.

Having discussed both the function of microglia and PIPs in neurodegenerative disorders, the next section will explore the potential outcomes of PIP dyshomeostasis on specific microglial functions. The functions covered are TLR signaling, purinergic signaling, endocytosis, chemotaxis, and migration.

## PIP Effects on Microglial Functions and Implications for Neurodegeneration

### Role of PIPs in TLR Signaling

#### TLR Signaling in Microglia

Toll-like receptors (TLRs) recognize conserved pathogen-associated molecular patterns (PAMPs) of bacteria, viruses, yeast, fungi, and parasites (Takeuchi et al., 2002). The human genome encodes 9 TLRs (TLR1–9), all of which are expressed in microglia (Bsibsi et al., 2002; Olson and Miller, 2004; Zhang et al., 2013). Within the brain, TLR expression is highest in glial cells (Lehnardt et al., 2002; Babcock et al., 2006), further demonstrating the importance of these signaling pathways regarding microglial function. TLR2 and 4 have been most studied in and appear to have the most relevance regarding neurodegenerative disease (Azam et al., 2019).

Upon activation, TLRs dimerize and recruit toll/interleukin 1 (TIR)-domain-containing adaptor proteins. These adaptor proteins are myeloid differentiation primary response protein 88 (MyD88, TLRs 1–2 and 4–9), TIR-domain containing adaptor protein (TIRAP, TLR 2 and 4), TIR domain-containing adaptor-interferon β (TRIF, TLR3 and 4), and TRIF-related adapter molecule (TRAM, TLR4; Takeda et al., 2003; Yamamoto et al., 2003a; Le et al., 2014). Following activation, MyD88 allows the nuclear translocation of NF-κB *via* recruitment of tumor-necrosis-factor-receptor-associated-factor 6 (TRAF6) and members of the IL-1R-associated kinases (IRAK) family. This results in proinflammatory cytokine and cyclooxygenase-2 (COX-2) production (Zhang et al., 1999; Takeda and Akira, 2004; Broad et al., 2007; Kawai and Akira, 2010). TLR-mediated TRIF and TRAM activation result in the induction of interferon-inducible genes, NF-κB dependent signaling pathways, and chemokine production (Schafer et al., 1998; Fitzgerald et al., 2003; Melchjorsen and Paludan, 2003; Yamamoto et al., 2003b; Pålsson-McDermott and O’Neill, 2004). In addition to the activation of inflammatory pathways, TLR signaling has been suggested to enhance phagocytosis (Tricker and Cheng, 2008).

#### Roles of Phosphoinositides in TLR Signaling

Phosphoinositides, in particular PI(4,5)P_2_, have been shown to act as key regulators of TLR4 signaling (Le et al., 2014). TLR4 is important in the immune response to LPS, heat-shock proteins, extracellular matrix proteins, and various neurodegeneration-related protein aggregates (Azam et al., 2019). Activation and subsequent dimerization of TLR4 induce the formation of a MyD88 and TIRAP protein complex (Yamamoto et al., 2002). TIRAP requires PI(4,5)P_2_ binding to its N-terminal region to initiate translocation to the plasma membrane, allowing for downstream signal transduction and cytokine production (Kagan and Medzhitov, 2006). This activation also results in increased PI(4,5)P_2_ at the plasma membrane (Kagan and Medzhitov, 2006), and conversely depleting PI(4,5)P_2_ stifles downstream TLR4 signaling (Wan et al., 2010). In primary microglia, BV2 microglia-like cells and primary astrocytes, this appears to occur *via* upregulation of the PI(4,5)P_2_ synthesis enzyme PIP-5 kinase following TLR4 activation (Jou et al., 2006; Lee et al., 2010a, b; Nguyen et al., 2013). In this way, phosphoinositide dyshomeostasis in neurodegenerative conditions could have substantial effects on TLR activity.

In addition to modulating adaptor protein localization, PI(4,5)P_2_ can also have indirect effects on TLR signaling. Activated TLR4 is subsequently internalized *via* clathrin- and dynamin-mediated endocytosis, where it initiates further downstream signaling pathways within early endosomes (Kagan et al., 2008). Endocytosis is tightly regulated by plasma membrane PI(4,5)P_2_ levels (Bohdanowicz and Grinstein, 2013), and studies have demonstrated how PI(4,5)P_2_ degradation by PLCγ2 is critical for TLR4 endocytosis (Zanoni et al., 2011; Schappe et al., 2018). In this way, changing PI(4,5)P_2_ levels in the context of various neurodegenerative disorders will affect TLR4 internalization and consequent signaling activation.

Furthermore, TLR9 can be seen to induce autophagosome/lysosomal fusion—a key event in autophagy—*via* the PI(4,5)P_2_ phosphatase oculocerebrorenal syndrome of lowe (ORCL). TLR9 signaling activates ORCL in lysosomes, which in turn reduces PI(4,5)P_2_ levels. PI(4,5)P_2_ is an inhibitor of TRPML1: an ion channel responsible for autophagy induction (De Leo et al., 2016). *Via* this mechanism, TLR9 dyshomeostasis, which has been observed in several neurodegenerative diseases including AD and PD (Fiebich et al., 2018), could result in altered PI(4,5)P_2_ levels, and perhaps result in autophagy dyshomeostasis. Substantial evidence demonstrates autophagy dysregulation in both AD and PD (Liu and Li, 2019; Hou et al., 2020).

#### TLR Signaling Within the Neurodegenerative Disease

Aging, a key risk factor for numerous neurodegenerative diseases (Hou et al., 2019), results in TLR dysregulation, characterized by both impaired signaling and inappropriate activation (Shaw et al., 2011). TLRs have highly established roles in numerous neurodegenerative conditions (Fiebich et al., 2018). For one, TLRs are upregulated in AD (Liu et al., 2005), ALS (Casula et al., 2011; Lee et al., 2015), and PD brains (Kouli et al., 2019). Furthermore, within human and mouse AD brains, upregulation of TLRs (TLR 2, 4, 5, 7, 9) has been observed within microglia surrounding amyloid plaques (Liu et al., 2005; Walter et al., 2007; Jana et al., 2008; Letiembre et al., 2009). Also, neurodegeneration-related proteins like Aβ (Jana et al., 2008; Richard et al., 2008; Caldeira et al., 2017) and α-synuclein (Beraud et al., 2011; Daniele et al., 2015) have been demonstrated to increase microglial TLR expression.

When closely examining links between TLRs and neurodegenerative disease, it quickly becomes apparent that the relationship between signaling and pathology is often complex. Increasing or decreasing the expression of various TLRs can have both protective and detrimental outcomes in a wide variety of neurodegenerative conditions (Rietdijk et al., 2016; Azam et al., 2019).

The importance of TLR signaling in preventing the development of AD is highlighted by studies demonstrating how activation of TLR 2, 4, and 9 signaling can reduce brain pathology and plaque build-up (Tahara et al., 2006). Furthermore, TLR 4 and 2 knock-out mice demonstrate increased amyloid plaque burden and cognitive decline (Song et al., 2011; Zhou et al., 2019). The above affects appear to occur *via* reduced microglial phagocytosis of amyloid plaques. Conversely, TLR4 polymorphisms which reduce receptor signaling have been observed to be protective against LOAD (Minoretti et al., 2006). Moreover, numerous studies have demonstrated how downregulating TLR signaling (2, 4, 6) can protect against AD development (Chen et al., 2016; Rietdijk et al., 2016; Zhang et al., 2016; Rangasamy et al., 2018; Long et al., 2019). The observed discrepancies in outcome when modulating TLR signaling in AD may reflect differences in disease progression at the time of treatment. TLR signaling inhibition to prevent excess neuroinflammation may be more effective at later stages of the disease, whilst activation to prevent the initial build-up of amyloid plaques appears to be beneficial at earlier stages (Go et al., 2016; Pourbadie et al., 2018).

Numerous studies demonstrate upregulated TLR signaling within PD. TLR upregulation within the PD brain is suspected to be responsible for the observed α-synuclein-induced microglial activation (Kouli et al., 2019). Within the HD field, however, so far only one study has examined the role of TLR signaling in disease pathogenesis. This study demonstrated how homozygous deficiency of TLR2 or 3 or heterozygous deficiency of TLR4 was able to extend lifespan in an HD mouse model (Griffioen et al., 2018). Although preliminary, this data suggests that further research into TLR signaling in HD, and perhaps investigating TLR inhibitors, would be a promising research avenue. In contrast to observations in PD and HD, TLR signaling in ALS appears to slow disease progression, with myD88 KO/ALS mice showing accelerated disease onset and reduced survival (Kang and Rivest, 2007). Another study demonstrated the importance of the TRIF pathway in protecting motor neurons within the ALS brain (Komine et al., 2018). These observations suggest that perhaps activating TLRs in ALS could provide some therapeutic benefit.

#### Targeting TLRs to Treat Neurodegenerative Disease—Focus on TLR4/PI(4,5)P_2_

Having summarized the well-characterized roles of TLRs and phosphoinositols in neurodegenerative conditions, the next question is whether we can exploit this knowledge when considering potential therapeutics. As TLRs have been implicated in the pathology of numerous diseases, both neurodegenerative and otherwise, many studies have characterized the effects of both natural and synthetic TLR agonists and antagonists (Gambuzza et al., 2014; Ain et al., 2020). Given the particular importance of phosphoinositides, namely PI(4,5)P_2_, in TLR4 signaling, this review will focus on TLR4 as a therapeutic target for neurodegenerative disease.

TLR4 activation to increase engulfment of misfolded protein could act as a promising treatment strategy within the early-AD brain. Potential candidates to activate TLR4 include the non-toxic LPS derivative monophosphoryl lipid A (Yousefi et al., 2019). As previously discussed, during later disease stages it is likely that inhibiting effects of TLR4 can protect against further neurodegeneration. Potential TLR4-pathway inhibitors that have shown promise against early AD phenotypes include the omega-3-polyunsaturated fatty acid alpha-linolenic acid (Ali et al., 2020), geniposidic acid (Zhou et al., 2020), and Alpinia oxyphylla-Schisandra chinensis (Qi et al., 2019). TLR4 inhibitors have also been investigated to treat PD. One such compound is vinpocetine, which appears to reduce TLR expression and improve the cognition of PD patients; although whether or not this improvement occurs specifically *via* effects on TLR signaling is yet to be determined (Ping et al., 2019).

As TLR signaling, particularly TLR4 signaling, is highly influenced by changing PI(4,5)P_2_levels, it may be possible to boost any protective effects by combining TLR-targeting and PI(4,5)P_2_ manipulating compounds. This could involve co-treating with drugs to increase PI(4,5)P_2_ levels when activating TLR4 and reducing PI(4,5)P_2_ when inhibiting TLR4. There are several options available for manipulating PI(4,5)P_2_ levels (Idevall-Hagren and De Camilli, 2015). One way would be by activating or inhibiting PLCγ2: the enzyme that breaks down PI(4,5)P_2_.

To conclude this section, TLR signaling, a key function of microglia, is dysregulated in numerous neurodegenerative conditions. This potentially allows for the possibility of using the same drug to treat multiple disorders. TLR signaling has strong links to phosphoinositide metabolism, another function known to be disrupted in the same conditions. These links could be exploited when investigating potential therapeutics.

### Roles of PIPs in Purinergic Signaling

#### Purinergic Signaling in Microglia

The purinergic signaling system has wide-ranging implications for CNS function. This system consists of enzymes, transporters, receptors, and other proteins which facilitate the recognition, secretion, and degradation of extracellular nucleotides and nucleosides. Within the CNS, nucleotides [such as adenosine triphosphate (ATP), adenosine diphosphate (ADP), and uridine diphosphate (UDP)] are released from cells in exosomes. ATP is often released from damaged cells following CNS injury (Neary et al., 1994). Following the release, nucleotides are rapidly degraded by ectonucleotidases, generating both other nucleotides and adenosine. Adenosine binds to P1 purinergic receptors (A1, A2A, A2B, and A3), which are widely expressed across the CNS. Adenosinergic signaling within microglia has key roles regarding activation. Nucleotides bind to ionotropic P2X(P2X1–7) and metabotropic P2Y (P2Y1, 2, 4, 6, 11–14) receptors, which are again widely expressed, and act as key mediators in neuronal-glial signaling networks. ATP binding to P2X receptors opens a non-selective Na^+^, K^+^, and Ca^2+^ cation pore. P2Y receptors are activated by a variety of nucleotides and share the seven-transmembrane-domain topology of G-protein coupled receptors. Activated P2Y 1, 2, 4, 6, and 11 receptors use G_q_/G_11_ to activate PLC and initiate Ca^2+^ release from the ER, which in turn induces store-operated Ca^2+^ entry *via* Orai1 and TRPC (Lim et al., 2017). P2Y 12–14 couple to G_i_/_0_, which activate G protein-gated, inwardly rectifying potassium (GIRK) channels to modulate downstream ion channels (Abbracchio et al., 2009; Erb and Weisman, 2012).

Microglia express the P1 receptors A1, A2A and A3 (Haskó et al., 2005), and the P2 receptors P2X4, P2X7, P2Y6, P2Y12, and P2Y13 (Calovi et al., 2019). A1R expression on microglia appears to reduce activation; A2AR expression occurs in response to immune-stimuli, results in cytokine and nitric oxide (NO) release, and affects neuronal survival; A3R expression promotes chemokine release (Boison et al., 2010). Increased P2X7R expression in microglia leads to microgliosis, NO and reactive oxygen species release, ATP release, NLRP3 inflammasome assembly, caspase-1 cleavage, chemokine, and cytokine release (Choi et al., 2007; Takenouchi et al., 2009; Shieh et al., 2014; He et al., 2017; Munoz et al., 2017; Yue et al., 2017). Persistent P2X7 activation also leads to the formation of a large non-selective pore that appears to reduce microglial viability and increase cytotoxicity (Seeland et al., 2015; Monif et al., 2016). Effects of P2X4R expression in microglia are not particularly well understood, although it also appears to promote activation and inflammation (Calovi et al., 2019). Furthermore, prolonged P2X4 activation appears to result in a large non-selective pore in a similar way to P2X7, although this pore appears non-cytotoxic (Bernier et al., 2012a). P2Y12R is established as a marker for healthy, ramified microglia (Mildner et al., 2017), is downregulated during activation (Haynes et al., 2006), and has key roles in cell migration and chemotaxis (Ohsawa et al., 2010). The roles of P2Y12R in chemotaxis will be further explored in a later section. Moreover, P2Y12R, alongside P2Y13R, have roles in inflammatory cytokine production and release from microglia (Liu et al., 2017). P2Y6, *via* UDP, initiates microglial phagocytosis (Neher et al., 2014), whilst also promoting neuroinflammation *via* cytokine (Yang X. et al., 2017), chemokine (Kim et al., 2011; Morioka et al., 2013), and NO production (Quintas et al., 2014).

The above information demonstrates the crucial role of purinergic signaling regarding a wide variety of microglial functions.

#### Roles of Phosphoinositides in Purinergic Signaling

All known P2X channels (except P2X5) have been demonstrated to be regulated by phosphoinositide signaling, with PIPs proving crucial cofactors for channel activity (Bernier et al., 2013b). This review will discuss in detail only P2X4 and P2X7 regulation by PIPs, as these are the P2X channels expressed in microglia (Calovi et al., 2019).

PI(4,5)P_2_ and PI(3,4,5)P_3_ have been demonstrated to increase P2X4 channel activity. Activity, including P2X4-mediated Ca^2+^ entry, can be stopped by depleting either PI(4,5)P_2_ or PI(3,4,5)P_3_ and rescued by intracellular injection of these lipids (Bernier et al., 2008). As previously mentioned, prolonged ATP-mediated P2X4 stimulation leads to the formation of a highly permeable pore, and this process is also inhibited by PI(4,5)P_2_ depletion (Bernier et al., 2012a). PIP binding appears to affect P2X activity by inducing a conformational change that affects channel gating (Bernier et al., 2008, 2012b). Specific lysine residues of the P2X4 C-terminal region appear crucial for PIP-P2X4 interactions, with mutation of these residues inhibiting PI(4,5)P_2_ and PI(3,4,5)P_3_ binding. The seemingly non-specific nature of the P2X binding site within the P2X4R means that it is likely regulated by a host of PIP species (Bernier et al., 2013b).

Pharmacological inhibition of PI(4,5)P_2_ synthesis has been demonstrated to reduce P2X7R current density (Zhao et al., 2007). Similar to P2X4, specific positively charged residues within P2X7R were found to be directly responsible for this PI(4,5)P_2_ mediated receptor activation (Zhao et al., 2007), although in this case interactions may be indirect (Bernier et al., 2012b). Indirect interactions of PI(4,5)P_2_ and other PIP-sensitive receptors *via* linker proteins has previously been characterized, and P2X7R has been shown to interact with α-actinin (Kim et al., 2001): a known linker protein which facilitates the interaction between PI(4,5)P_2_ and glutamate receptors (Kim et al., 2008).

Increasing evidence suggests the PI(4,5)P_2_ degradative enzyme PLCγ2 as an indirect regulator of numerous P2X channels *via* modulation of PI(4,5)P_2_ levels (Bernier et al., 2013b). This enzyme-driven channel regulation *via* PIP synthesis/degradation has been demonstrated for several other types of receptor, for example, PIP degradation by PLC modulates TRPM7, GIRK, and KCNQ channel activity (Caulfield et al., 1994; Kobrinsky et al., 2000; Runnels et al., 2002; Cho et al., 2005; Brown et al., 2007). Moreover, stimulating PLCγ2-mediated PI(4,5)P_2_ hydrolysis *via* activation of platelet-derived growth factor receptor led to reduced P2X7R activity, with PI(4,5)P_2_ addition reversing this effect in macrophages (Zhao et al., 2007). This theory is further supported by observations that UDP-mediated activation of P2Y6 leads to PLC activation within microglia, followed by reduced P2X4R activity, presumably due to falling PI(4,5)P_2_ levels (Bernier et al., 2013a). As P2Y receptors often signal *via* PLCγ2, which is in turn regulated by PI(4,5)P_2_ levels (Erb and Weisman, 2012), P2Y signaling is also likely to be tightly linked to phosphoinositide homeostasis within microglia.

In addition to interaction with P2X channels, ATP and PI(4,5)P_2_ binding has been demonstrated to co-regulate key intracellular signaling proteins. This includes focal adhesion kinase, which has been demonstrated to impact microglial mobility (Choi et al., 2015). Furthermore, ATP-sensitive potassium channels or K_ATP_ channels, which have key roles regarding initiation of inflammation by microglia (Rodriguez et al., 2013), are also co-regulated by PI(4,5)P_2_ binding (Li et al., 2017).

The above evidence demonstrates a clear regulatory function of phosphoinositide species, particularly PI(4,5)P_2_, with regards to purinergic signaling. This means that PIP dyshomeostasis within neurodegenerative disease will likely have substantial implications regarding microglial purinergic signaling and downstream phenotypes.

#### Purinergic Signaling and Neurodegenerative Disease

Purinergic signaling has well-established roles within numerous neurodegenerative disorders including AD, PD, HD, and ALS (Puchałowicz et al., 2014).

P1 receptors are seen to be upregulated early in disease progression within the most affected areas of PD patient brains (Villar-Menéndez et al., 2014). Also, two polymorphisms in the A2A receptor appear to reduce PD risk (Popat et al., 2011). This dysregulation is also seen in AD, with increased A2A receptor expression observed in the cortex and hippocampal microglia in post-mortem brains (Angulo et al., 2003). Following these observations, many clinical trials are currently underway investigating A1 receptor antagonists as a therapeutic target for Parkinson’s disease, with some showing promise (Tóth et al., 2019). *In vitro* and *in vivo* studies suggest similar neuroprotective effects of A1 modulators in AD, with antagonism of A2ARs appearing to reduce amyloid plaque formation (Woods et al., 2016). One such A2A receptor antagonist is caffeine (Fredholm et al., 1999): known to reduce the risk of a variety of neurodegenerative conditions (Eskelinen and Kivipelto, 2010). Within ALS however, activation of A2A receptors appears protective, with caffeine negatively affecting neurological phenotypes, although this effect is stage-dependent (Sebastião et al., 2018). A similar effect, alongside downregulation of the A2A receptor, is seen in HD (Blum et al., 2018).

P2X receptors are also acknowledged to play important roles in neurodegenerative disease development. P2X7R is the most widely studied regarding its roles in neurodegeneration, following observations that it is upregulated in microglia from a variety of conditions including AD and PD (McLarnon et al., 2006; Durrenberger et al., 2012). Within AD, increased microglial purinergic signaling *via* the P2X7R appears to contribute to both altered Aβ metabolism, a heightened inflammatory response, and synaptotoxicity (Woods et al., 2016). Though not as well studied as P2X7, P2X4 also appears to be dysregulated in the AD brain and is seen to increase following exposure to Aβ (Varma et al., 2009). These observations, alongside known roles of the P2X4R in eliciting inflammatory responses (Calovi et al., 2019), suggest potential roles in AD pathology. Within PD, P2X7 receptors are thought to contribute to pathology *via* increased synaptotoxicity, neurotoxicity, and gliosis (Carmo et al., 2014). Though current research on purinergic signaling in PD has focused on P2X7, P2X4 has also been implicated in PD pathology. Altered P2X4 signaling in PD is thought to interfere with dopaminergic signaling, therefore contributing to observed difficulty with motor control and sensorimotor gating in PD mouse models (Khoja et al., 2016). P2X7R expression is also increased within HD mouse brains, and treating with a P2X7R antagonist inhibits neuronal loss while improving motor coordination (Díaz-Hernández et al., 2009). Within ALS, in contrast to that observed in AD, PD, and HD, P2X4, and P2X7 expression appear to protect against neurodegeneration (Oliveira-Giacomelli et al., 2018). Indeed, allosteric P2X4 activation increases the lifespan of ALS-mice (Andries et al., 2007) and P2X7R knock-out ALS mice show accelerated neurodegeneration (Apolloni et al., 2013).

P2Y receptors are also dysregulated in a variety of neurodegenerative conditions. Within AD microglia, P2Y signaling can be seen to affect microglial migration, chemokine and cytokine production, endocytosis, phagocytosis, Aβ metabolism, and oxidative stress responses (Erb et al., 2015). UDP-mediated P2Y6 signaling appears to increase phagocytosis of viable neurons by activated microglia. Perhaps unsurprisingly given the above observations, the P2Y6R has been suggested to contribute to microglial activation, inflammation, and phagocytosis of viable neurons in PD. P2Y6R appears to increase in PD models, and antagonists of this receptor appear to delay neuronal death following inflammation (Yang X. et al., 2017; Oliveira-Giacomelli et al., 2019). To our knowledge, no research has been done currently investigating UDP/P2Y6R signaling in AD models, although this would likely be a fruitful avenue in future studies. P2Y12, alongside its roles in chemotaxis, has been suggested to be important for synaptic plasticity and synaptic pruning (Sipe et al., 2016). As increased synaptic pruning is emerging as a key phenotype of neurodegenerative diseases, including AD and PD, this receptor may have roles in the pathology of a variety of neurodegenerative disorders (Lee and Chung, 2019). Microglial P2Y13R expression has been shown to induce astrocyte proliferation, which could also have implications for neurodegeneration, although no studies have specifically looked at this in this receptor context of neurodegenerative diseases (Quintas et al., 2018).

Whist more work needs to be done to thoroughly characterize the roles of microglial purinergic signaling in neurodegenerative disease, these signaling pathways have clear implications regarding dementia pathology.

#### Targeting Purinergic Signaling to Treat Neurodegenerative Disease

Drugs targeting purinergic signaling are showing great promise regarding the treatment of a variety of neurodegenerative disorders.

Several A2A receptor antagonists have been characterized and investigated in clinical trials on PD patients, showing various degrees of success. So far, however, despite the benefits of A2A antagonism seen *in vivo* and *in vitro* in AD models, no compounds have moved to clinical trials as of yet for this condition. Several P2Y receptor antagonists exist, although to date none have been investigated as potential treatments for neurodegenerative diseases (Von Kügelgen and Hoffmann, 2016). A variety of A2A receptor agonists are available, although many appear to have adverse side effects (Guerrero, 2018).

P2X7 antagonists appear to reduce pathologies in several neurodegenerative disorders, including AD and PD (Burnstock and Knight, 2018). Many have been previously tested in clinical trials for the treatment of non-neurological disorders, such as rheumatoid arthritis and Crohn’s disease (Cao et al., 2019). Following its promise as a potential wide-ranging therapeutic target, numerous highly potent, stable, centrally penetrant P2X7R antagonists are currently being developed and tested for a wide range of conditions (Rech et al., 2016). P2X4 antagonists have proved harder to generate, although some are now available and are likely to be investigated in future studies for beneficial effects on neurodegenerative disorders (Stokes et al., 2017).

Given the potential of P2XR7 and P2XR4 inhibition as potential therapeutic targets for neurodegenerative disorders, and that these channels are activated by PIPs, it may be the case that dual P2X and PIP synthesis inhibition could act as a potential therapeutic. As mentioned in the previous section on TLR signaling, there are several options available for modulating PIP levels (Idevall-Hagren and De Camilli, 2015). Moreover, given that PLCγ2 hydrolysis of PI(4,5)P_2_ can be seen to modulate P2X activity (Bernier et al., 2013b), potentially activating PLCγ2, alongside direct purinergic signaling modulation, could be protective against neurological disease progression.

In summary, purinergic signaling, a key regulator of microglial function, is dysregulated in numerous neurodegenerative disorders. This process has strong links to phosphoinositide metabolism. These links could be exploited when investigating potential therapeutics.

### Role of PIPs in Microglial Endocytosis

#### Endocytic Systems in Microglia

Microglia, like all tissue-resident macrophages, are dedicated phagocytes tasked with immune surveillance and the elimination of pathogens. These cells can recognize, engulf and destroy foreign bodies. In addition to their immuno-protective role, microglia also perform important housekeeping tasks such as removing apoptotic cells and mediating synaptic pruning during development (Wake et al., 2011). Microglia survey their environment by constantly patrolling a set region for indications of danger or damage (Madry et al., 2018). Large particles are internalized *via* phagocytosis, whilst other endocytic uptake methods (e.g., micro and macro-pinocytosis) are utilized for the uptake of fluid-phase material and soluble antigens (Solé-Domènech et al., 2016). Micropinocytosis includes receptor-mediated uptake methods, such as clathrin-mediated and caveolae-mediated endocytosis (Mettlen et al., 2018; Li et al., 2019).

During phagocytosis and macropinocytosis, large vacuoles are known as phagosomes, and macropinosomes form *via* actin rearrangement (May and Machesky, 2001). These vacuoles engulf the target material following the invagination of the plasma membrane. The vacuole is then brought into the cytoplasm where it fuses with the lysosome, which degrades the captured material (Gray et al., 2016). Phagocytosis and macropinocytosis can be artificially divided into two phases. First the formation of the vacuole, and then its progression and maturation through the endocytic pathway. Similar molecular machinery is involved in phagocytic and macropinocytic systems. These processes involve complex signaling cascades, which lead to cytoskeletal reorganization and membrane remodeling. Both systems begin with Rho GTPase activation and the extension of actin-driven membrane protrusions (West et al., 2000), followed by activation of PI3K which results in large-scale membrane remodeling (Araki et al., 1996).

Clathrin-mediated endocytosis is the major entry route for extracellular hormones and signaling factors and serves to regulate the internalization of transmembrane receptors as well as the recycling of pre-and postsynaptic membrane proteins (Le Roy and Wrana, 2005). Caveolae are invaginations of the plasma membrane generated by caveolins, proteins with a membrane-integral hairpin anchor, and cavins, cytoplasmic proteins that are required for the stabilization of caveolae (Parton and del Pozo, 2013). Following their internalization, caveolae display multiple additional roles within the cell, participating in mechano-sensing, compartmentalized signaling, and lipid metabolism (Del Pozo et al., 2020).

The following sections discuss the role of PIPs in the above described endocytic processes and speculated involvement of these lipids is summarized in Table 3.

**Table 3:** Suspected involvement of PIP species in various forms of endocytosis.

Type of endocytosis	PIP species involved	Mechanism	References
Phagocytosis	PI(4,5)P_2_	Increase following target recognition allows the formation of pseudopodia. Later reduction essential for the completion of phagocytosis.	Coppolino et al. (2002) and Scott et al. (2005)
	PI(3)P	The transient increase allows maturation and sealing of phagosomes.	Vieira et al. (2001)
Macropinocytosis	PI(4,5)P_2_	Enriching this PIP in membrane ruffles stimulates macropinocytosis.	Donaldson (2003)
	PI(3)P	Participates in vacuole formation.	Yoshida et al. (2009)
Clathrin-mediated endocytosis	PI(4,5)P_2_	Required for the invagination of clathrin-coated vesicles.	Antonescu et al. (2011)
Caveolae-mediated endocytosis	PI(4,5)P_2_	Accumulates at the rim of caveolae vesicles.	Nunes and Demaurex (2010)

#### Roles of Phosphoinositides in Phagocytosis

When microglia initially encounter phagocytic targets, extracellular signals must be conveyed across the plasma membrane to initiate the complex cellular behaviors that culminate in uptake. It is becoming increasingly apparent that PIPs play a prominent role in relaying this information. Indeed, both the detection of ligands by transmembrane phagocytic receptors and the ruffling of membranes during macropinocytosis are accompanied by local changes in PIP composition (Gillooly et al., 2001). PIPs also coordinate phagosome maturation, whereby membrane fusion and fission events lead to the acquisition of degradative properties (Vieira et al., 2002).

**Figure 2:**
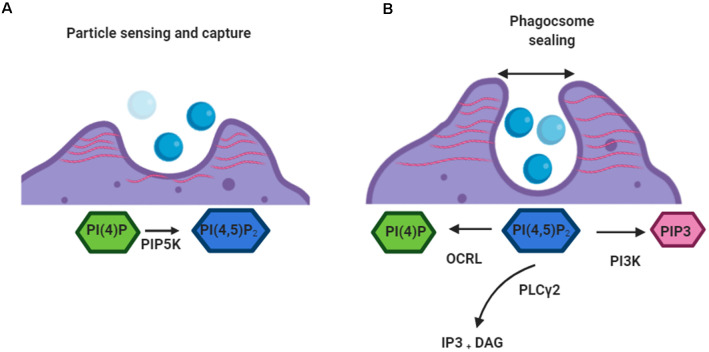
Roles of PI(4,5)P_2_ in early phagocytosis.** (A)** When a target is detected by a phagocytic cell PI(4)P is converted to PI(4,5)P_2_by PIP5K. PIP5K associates with the plasma membrane along its positively charged surface. PI(4,5)P_2_ mediates linkage of actin networks (red) to integral plasmalemmal proteins through intermediary ezrin, radixin, and moesin (ERM) proteins. **(B)** When the phagosome sealing begins depletion of PI(4,5)P_2_ from the base of the cup leads to the removal of actin filaments. PI(4,5)P_2_ is converted by kinases (PI3K), phosphatases (OCRL), and phospholipases (PLCγ). This allows the movement of the closed vacuole into the cell.

PI(4,5)P_2_ and its metabolites (Figure 2) are pivotal to the control of numerous events in phagocytosis including the rearrangement of the actin cytoskeleton (Rohatgi et al., 2000), receptor mobility (Jaumouillé and Grinstein, 2011), integrin activation (Martel et al., 2001), and ion channel activity (Suh and Hille, 2005). To control these disparate events it is important that PI(4,5)P_2_ levels change only locally during phagocytosis and that each event occurs in discrete locations (Kutateladze, 2010). Work by Botelho et al. (2000) characterized an accumulation of PI(4,5)P_2_ in emerging pseudopods during the early stages of phagosome formation, followed by a drop in levels at the base of the phagocytic cup as the pseudopodia extend. Following phagosome sealing and severing, phagosomal PI(4,5)P_2_ decreases precipitously and is no longer detectable by fluorescence microscopy (Botelho et al., 2000). This decrease appears to initially occur *via* PI3K-mediated phosphorylation of PI(4,5)P_2_ into PI(3,4,5)P_3_. PI3K also serves as a signal for the recruitment of PLCγ, which then acts as the predominant method for reducing PI(4,5)P_2_levels within phagosomes (Falasca et al., 1998).

PI(4,5)P_2_ promotes the activation of several actin-regulatory proteins which encourage filament assembly and inhibit disassembly (Saarikangas et al., 2010). This leads to an increase in the number of barbed ends and also induces *de novo* actin nucleation by activating nucleation-promoting factors (Miki et al., 1996). Also, ezrin, radixin, and moesin (ERM) proteins, which link the cytoskeleton to the plasma membrane, are known to be partially controlled by PI(4,5)P_2_ levels (Bretscher et al., 2002). As such the localized increase in PI(4,5)P_2_ that occurs after the activation of phagocytic receptors results in reorganization of the actin cytoskeleton, driving the extension of pseudopodia around the surface of phagocytic targets (Coppolino et al., 2002). Blocking this local increase in PI(4,5)P_2_ appears to prevent the formation of phagocytic cups and therefore inhibits phagocytosis (Coppolino et al., 2002). Interestingly, dismantling of actin at the base of the cup and particle internalization are also blocked if high PI(4,5)P_2_ levels are sustained by promoting PIP5K-mediated synthesis or by inhibiting PLCγ-driven degradation (Scott et al., 2005). This suggests that the reduction of PI(4,5)P_2_ is linked to actin disassembly, which is in turn required for the completion of phagocytosis.

In addition to the consequences that PI(4,5)P_2_ metabolism has on cytoskeletal dynamics, the breakdown of this PIP to its secondary metabolites also has important ramifications in the phagocytic process. PLCγ-mediated hydrolysis of PI(4,5)P_2_ leads to the formation of DAG and Ins(1, 4, 5)P_3_ (IP_3_). DAG generation coincides in space and time with the disappearance of PI(4,5)P_2_. Interestingly, though neither DAG nor IP_3_ is essential for particle engulfment, inhibition of PLCγ blocks the phagocytic response (Botelho et al., 2000; Scott et al., 2005). This suggests that it’s the disappearance of PI(4,5)P_2_, rather than the formation of its metabolites that is required for phagocytosis. However, DAG recruitment of PKC isoforms and increased Ca^2^ levels in the cytoplasm is required for later stages of phagocytosis (Ueyama et al., 2004; Nunes et al., 2012; Schlam et al., 2013; Bengtsson et al., 1993).

Like other 3-polyphosphoinositides, PI(3,4,5)P_3_ levels are scarce in unstimulated cells. However, PI(3,4,5)P_3_ is quickly generated following activation of immune receptors. The metabolism of PI(3,4,5)P_3_ is strictly and dynamically regulated, and in general restricted to the cytosolic side of the cell membrane (Palmieri et al., 2010). During phagocytosis, the spatiotemporal dynamics of PI(3,4,5)P_3_ synthesis mirror those of PI(4,5)P_2_ breakdown, consistent with a role for class I PI3K in mediating the conversion of PI(4,5)P_2_ to PI(3,4,5)P_3_. PI3K is recruited to and activated at sites of phagocytosis following particle engagement (Marshall et al., 2001). Synthesis of phagosomal PI(3,4,5)P_3_ is detectable shortly after phagocytic targets are engaged, and this PIP continues to accumulate as the phagocytic cup progresses. While PI(3,4,5)P_3_ is still detectable after sealing, its presence in the phagosomal compartment is short-lived, and its concentration declines sharply within 1–2 min. Notably, SHIP accumulates at the phagosomal membrane (Marshall et al., 2001), where it promotes the breakdown of PI(3,4,5)P_3_ to PI(3,4)P_2_ (Marshall et al., 2001; Kamen et al., 2007). Perhaps unsurprisingly given its key and early role in the phagocytic process, near-complete inhibition of phagocytosis is seen in macrophages treated with PI3K inhibitors (Cox et al., 1999).

Though its cellular concentration is comparatively low, the PIP PI(3)P is also critically involved in the maturation of phagosomes. In mammalian cells, PI(3)P is found mainly at the cytoplasmic leaflet of early endosomes and in intraluminal vesicles of multivesicular bodies (Kaminska et al., 2016). However, sealing of the phagosome and its internalization is followed by a striking yet transient accumulation of this PIP, which lasts for about 10 min and coincides with the centripetal movement of the phagosomal vacuole (Vieira et al., 2001).

#### Roles of Phosphoinositides in Other Forms of Endocytosis

A central role for PIPs as spatial landmarks for membrane trafficking in other forms of endocytosis has emerged (Cremona and De Camilli, 2001; De Matteis and Godi, 2004). Despite only constituting <10 % of the total cellular phospholipids, PIPs act as key regulators of intracellular membrane traffic and cell signaling. Together with their corresponding vesicle adaptors and transmembrane cargo proteins, phosphoinositides can be seen as part of a system for directing membrane trafficking pathways (Wenk and De Camilli, 2004). PI(4,5)P_2_is required for the invagination of clathrin-coated vesicles (CCVs), the fusion of secretory granules with the plasmalemma, and for macropinocytosis. Other PIPs have been localized to distinct intracellular membranes and it now seems that many of the key proteins involved in vesicle formation, fusion, and fission are important targets of these lipids. For one, the formation of PI(4,5)P_2_-enriched membrane ruffles by overexpression of Arf6-GTP stimulates clathrin-independent macropinocytosis (Donaldson, 2003). Several enzymatic activities appear to contribute to PI(4,5)P_2_ degradation, which is required for the uncoating of CCVs (Cremona et al., 1999). Among these are PLCδ, inositol 5-phosphatases including synaptojanin, SHIP, OCRL, 5-phosphatase II, and proline-rich inositol polyphosphate 5-phosphatase. Moreover, PI(4,5)P_2_ is the precursor for PI(3,4,5)P_3_ synthesis following activation of PI3K by ligand-bound cell signaling receptors (De Matteis and Godi, 2004). How exactly the interplay between PI kinases and phosphatases is regulated is unclear.

Several studies have documented the presence of PI(3,4)P_2_ in macropinosomes. This PIP appears to be generated by SHIP2 and broken down by INPP4B (Hasegawa et al., 2011; Welliver and Swanson, 2012; Maekawa et al., 2014). PI(3)P can also be found within early macropinosomes in myeloid cells (Yoshida et al., 2009). Unlike during phagocytosis, where PI(3)P is believed to be important for vesicle maturation, during macropinocytosis PI(3)P has been proposed to participate in vacuolar formation. Additionally, inhibiting PI(3)P synthesis by knocking down INPP4B impairs micropinocytosis (Maekawa et al., 2014).

Whilst the core components of caveolae are not known to associate with PIPs directly, the dynamin-related ATPase EHD2 binds PI(4,5)P_2_-rich membranes before recruitment to caveolae containing vesicles (He et al., 2004). EHD2 functions as a negative regulator of caveolae internalization by retaining this protein at the plasma membrane and this function require lipid-binding (Cheng et al., 2007). Interestingly, Nunes and Demaurex (2010) labeled PI(4,5)P_2_ bound to the PH-domain of PLCδ on freeze-fractured plasma membrane leaflets and reported the accumulation of PI(4,5)P_2_ at the rim of caveolae vesicles. The precise significance of the role of PIPs in caveolin-mediated endocytosis thus remains elusive, yet caveolae do appear to be regulated by PI(4,5)P_2_.

#### Endocytosis and Neurodegenerative Disease

One of the principal roles of microglia in neurodegeneration is the clearance of protein aggregates, myelin debris, and apoptotic cells in an attempt to maintain healthy brain homeostasis. Many neurodegenerative conditions present with increasing accumulation of toxic extracellular proteins such as Aβ plaques in AD, and increased apoptosis of cells such as the loss of dopaminergic neurons in PD. It is therefore clear that alterations in microglial phago and endocytosis would have important implications regarding the progression of neurodegenerative conditions.

FcR-mediated phagocytosis and complement activation play a critical role in the removal of plaques from the AD brain (Lee and Landreth, 2010). Furthermore, monocyte chemotactic protein-1 (MCP-1/CCL2), coupled with its binding receptor CC-chemokine receptor 2, appear crucial mediators of the neuroinflammatory response that drives the disease process in a mouse model of AD (Kiyota et al., 2009, 2013; Bose and Cho, 2013). CCL2-deficient AD mice (APP/PSEN1 mice) showed decreased microglial phagocytosis of both monomeric and oligomeric Aβ42 and accelerate Aβ deposition (Kiyota et al., 2013). Moreover, GWAS studies (Sims et al., 2017) have found several AD disease risk factors that are linked to both phagocytosis and phosphoinositides including a hyper-functional protective variant of PLCγ2 which hydrolyzes PI(4,5)P_2_ (Magno et al., 2019). Knockout of PLCγ2 has been shown to reduce phagocytosis in microglia (Andreone et al., 2020). Similarly, AD-GWAS variants in TREM2, an upstream receptor in this pathway, have been shown to affect phagocytosis both positively and negatively (Kim et al., 2017). Abi3, another risk factor for AD, is linked to actin polymerization and may also have a role in phagocytosis (Moraes et al., 2017; Conway et al., 2018). It is important to note however that despite the observed protective roles of microglial phagocytosis in the neurodegenerative brain increasing phagocytosis in microglia may not always improve brain pathology. AD microglia have been shown to display disrupted microglia-mediated synaptic pruning, which correlates with decreased cognitive ability (Brucato and Benjamin, 2020).

In addition to observations in AD, *in vitro* studies of microglia treated with monomeric α-synuclein as a model of PD exhibit enhanced phagocytosis (Park et al., 2008). Using proteomic technology, Liu et al., have shown that a variety of types of membrane proteins were potentially involved in microglial internalization of α-synuclein (Liu et al., 2007). In particular, clathrin was demonstrated to play a critical role in the endocytosis of aggregated α- synuclein, probably in a receptor-ligand sequestration-related manner, although the exact mechanism requires further study (Liu et al., 2007). In Huntington’s disease extracellular mHTT is cleared by microglial phagocytosis (Crotti and Glass, 2015). Moreover, within FTD and ALS, mutations in phagocytosis-associated genes expressed by microglia in the CNS have been identified as risk factors. These genes include missense mutations in TREM2 (Cady et al., 2014; Kleinberger et al., 2014). Also, mutations in PFN1, encoding Profilin, have been identified as a causative mutation in ALS. Profilin is important for the regulation of actin dynamics (Wu et al., 2012; Fil et al., 2017).

#### Targeting Microglial Endocytosis to Treat Neurodegenerative Disease

As discussed in the previous section, microglial phagocytosis plays an important role in the neuroimmune response to neurodegenerative conditions. As such it presents a tempting target for therapeutic intervention. However, it is worth remembering one of the clinical symptoms of AD is the chronic loss of synapses caused by microglial phagocytic engulfment (McQuade and Blurton-Jones, 2019). Moreover, AD and PD microglia can be seen to contribute to neurodegeneration *via* phagocytosis of injured but functional neurons (Brown and Neher, 2012; McQuade and Blurton-Jones, 2019). As such simply upregulating phagocytosis may not be useful. Ongoing genetics-based studies however may suggest a more effective route allowing a more targeted approach. There is a significant amount of work to still be done in this area before any therapies can be brought to the clinic. Given the importance of PIPs throughout the phagocytic process, possibly the future therapeutics targeting phagocytosis would benefit from co-manipulation of PIP levels.

### Role of PIPs in Microglial Chemotaxis and Migration

#### Chemotaxis and Migration in Microglia

Cell migration is crucial to the function of microglia, allowing them to patrol their region of interest and respond to sites of damage. Microglia react rapidly to damage signals with a positive chemotactic response. Upon detection of these signals, microglia undergo complex molecular and cytoskeletal changes that polarize the cell towards the direction of the damage site. Once stimulated to migrate, cells form a coordinated outgrowth of protrusions and adhesions, which results in translocation of the cell body by contraction towards the adhering zones. Finally, the adhesions are disassembled and the rear of the cell is retracted (Smolders et al., 2019). As a cell advances, newly extended protrusions adhere to the extracellular substrate using integrins. Integrins are attached to interacting myosin II and actin filaments (F and G type) *via* adaptor proteins, which allows for the generation of traction force (Lauffenburger and Horwitz, 1996). Previous studies have demonstrated that primary rat microglia (P0–P2) do not demonstrate classic types of adhesions during migration (which uses cell adhesion molecules such as cadherins), but instead form podosomes. These are 0.4–1 μm multimolecular structures with an F-actin core surrounded by a ring of adhesion and structural proteins. Through Ca^2+^ signaling in these podosomes, microglia are able to adhere to and degrade fibronectin substrates using matrix metalloproteinases. This allows them to transverse the extra cellular matrix (Siddiqui et al., 2012; Vincent et al., 2012).

Microglia mobility can be broadly divided into two main functional modes; surveillance and chemotaxis. Both these systems involve altering the cytoskeletal structure of the microglia using the high amounts of filamentous actin in motile bundles present in microglial cells (Capani et al., 2001; Lambrechts et al., 2004). *In vivo* and *in situ* studies using genetically targeted microglia have demonstrated that microglial tissue surveillance in the healthy CNS is almost exclusively performed by their long, thin, and highly branched processes, which extend and retract at average velocities of 2.5 μm/min (Davalos et al., 2005; Nimmerjahn et al., 2005; Wu et al., 2007; Li et al., 2012). Their high process motility (as well as their high cell density in the brain) allows microglia to scan the entire brain parenchyma once every few hours (Nimmerjahn et al., 2005). The mechanism of this surveillance is not fully understood but is thought to be monitored in part *via* signaling by astrocytes (Cotrina et al., 2000; Xiong et al., 2018) and neurones (Nimmerjahn et al., 2005; Fontainhas et al., 2011; Li et al., 2012; Gyoneva and Traynelis, 2013; Dissing-Olesen et al., 2014) as well as by fractalkine signaling (Zujovic et al., 2000; Cardona et al., 2006; Liang et al., 2009). However, it has been demonstrated that unlike chemotaxis, surveillance is not regulated by P2Y12 receptors (Haynes et al., 2006).

Microglia detect damage (*via* activation of P2Y12 purinergic receptors or fibrinogen-sensing CD11b/CD18 receptors) and immediately extend processes toward the site of injury, where they converge in less than 30 min to form a spherical shield preventing further spread (Davalos et al., 2005; Hines et al., 2009). As previously mentioned in this review, *in vitro* studies have established a key role for extracellular nucleotides like ATP/ADP as potent inducers of microglial chemotaxis (Honda et al., 2001; Franke et al., 2007; Orr et al., 2009). These nucleotides, as well as other signals like NO, are known to leak from damaged cells and so act as a signal of damage (Neary et al., 1994). Microglia use ionotropic P2X and metabotropic P2Y and P1 receptors to respond to extracellular nucleotides and nucleosides (Haynes et al., 2006; Koizumi et al., 2007; Wu et al., 2007; Avignone et al., 2008; Orr et al., 2009). Within cultured rat microglia, increased membrane ruffling and chemotaxis upon ADP stimulation appears to occur *via* the purinergic P2Y12R. ATP/ADP-induced chemotaxis, dependent on Gi-coupled P2Y receptors, was first described in cultured microglia and later *in vivo* (Honda et al., 2001; Davalos et al., 2005), and knock-out of ADP-activated Gi-coupled P2Y12 greatly decreases chemotaxis (Haynes et al., 2006). Expression of the P2Y12 receptor on the surface of ramified microglia *in vivo* (Haynes et al., 2006) is particularly enriched at the tips of the leading processes during chemotaxis (Dissing-Olesen et al., 2014). ATP/ADP-induced P2Y12 receptor activation leads to PLC and Ca^2+^dependent phosphorylation of the serine/threonine kinase Akt, as well as PI3K-mediated Akt phosphorylation (Irino et al., 2008).

#### Role of Phosphoinositides in Chemotaxis and Migration

**Figure 3:**
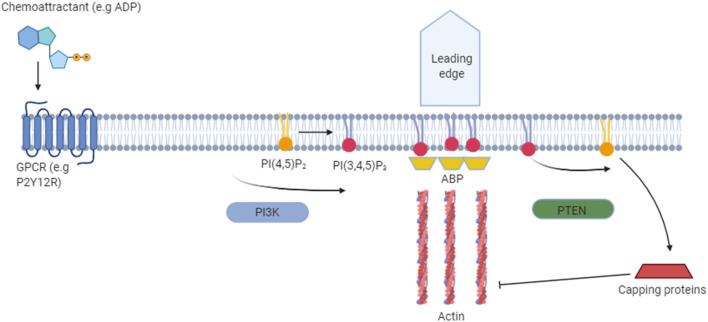
Functional role of phosphoinositides in cell migration. The binding of a chemoattractant to G-protein coupled receptors (e.g., P2Y12R) in the cell membrane releases the Gα heterodimer from the heterotrimeric Gα proteins. Dissociated Gα proteins stimulate PI(3,4,5)P_3_ production from PI(4,5)P_2_ *via* phosphoinositide 3-kinase (PI3K) and lead to membrane translocation of PI(3,4,5)P_3_-binding actin-binding proteins (ABPs) such as myosin. This allows remodeling of the actin cytoskeleton at the leading edge, which is required for the formation of novel cell protrusions. Away from the leading edge PI(3,4,5)P_3_ is converted back to PI(4,5)P_2_ *via* phosphatase and tensin homolog (PTEN). PI(4,5)P_2_ then inhibits actin assembly by binding capping proteins.

*In vitro* PI3K appears to act as one of the major signaling components of chemotaxis by allowing cells to establish polarity (Fan et al., 2017). PI3K is selectively localized at the leading edge of the membrane after exposure to a chemoattractant gradient. This creates a spatially restricted production of PI(3,4,5)P_3_ from PI(4,5)P_2_, which induces F-actin polymerization at the front of migrating cells (Parent et al., 1998; Haugh et al., 2000; Rickert et al., 2000). PTEN, which localizes away from the leading edge, acts reciprocally to PI3K by converting PI(3,4,5)P_3_ to PI(4,5)P_2_ (Wu et al., 2014; Figure 3).

P2Y12R has also been reported to be linked to a potassium channel, and ATP/ADP-induced activation of P2Y12R elicits an outward potassium current in microglia (Swiatkowski et al., 2016). Blocking this current abolishes chemotaxis to ATP, suggesting that this current plays an important role in the regulation of microglial motility.

Interaction with PIP species is crucial regarding actin assembly, with these lipids facilitating the crosslinking and linking of actin to the plasma membrane by binding with several different actin-binding proteins (ABPs; Figure 3). The ABP gelsolin is a key regulator of actin filament assembly and disassembly. Gelsolin caps to the barbed ends of G and F actin filaments, where it prevents monomer exchange (end-blocking or capping; Weeds et al., 1986), promotes nucleation (the assembly of monomers into filaments) and severs existing F-actins. Gelsolin binds PI(4,5)P_2_ and PI(3,4,5)P_3_ *in vivo* through two regions that contain clusters of basic residues. Overlapping binding sites means that PIP binding inhibits gelsolin from binding to the G/F-actin (Xian and Janmey, 2002). Gelsolin not only binds to charged regions on PIPs but also interacts with the fatty acid side chains and thus pulls out phospholipids from lipid bilayers. Through this mechanism, gelsolin may modulate PIP density in the plasma membrane (Liepina et al., 2003).

Cofilin proteins are a family of ABPs which are structurally and functionally related to gelsolin. These proteins bind to both G and F-actin and cause depolymerization at the minus end of filaments, thereby preventing their reassembly. Both PI(4,5)P_2_ and PI(3,4,5)P_3_ bind to cofilin and inhibit its activity (Ojala et al., 2001).

α-Actinin belongs to the spectrin gene superfamily. This protein connects actin filaments to integrins and serves as a scaffold to integrate signaling components at adhesion sites and promote bundling of actin filaments (Otey and Carpen, 2004). These proteins contain a PIP-binding site within the calponin homology domain (CH1 and CH2), close to the actin-binding site. α-actinin binds to both PI(4,5)P_2_ and PI(3,4,5)P_3_ with equal affinity. *In vivo* studies show that PI(3,4,5)P_3_ disrupts the connection between α-actinin and F-actin, although interestingly the opposite is seen in *in vitro* studies. Furthermore, the elevation of PI(3,4,5)P_3_ appears to disrupt the link between actin and integrins, which allows for the redistribution of focal adhesion points in migrating cells (Fraley et al., 2005).

The Ezrin/radixin/moesin (ERM) protein family provides a regulated linkage between the plasma membrane and the underlying actin cytoskeleton (Tsukita and Yonemura, 1997). Several studies have indicated that the binding of ERM proteins to PI(4,5)P_2_ and phosphorylation of a threonine residue in the F-actin binding site causes the dissociation of activated ERM proteins (Crepaldi et al., 1997; Naba et al., 2008).

Septins are a group of highly conserved GTP-binding proteins that assemble into filaments and are increasingly recognized as a crucial component of the cytoskeleton (Mostowy and Cossart, 2012). Septins act as a scaffold, allowing the recruitment of many proteins. *In vitro* studies have shown that purified septins bind phospholipids and that they display particular affinities for PI(4,5)P_2_ and PI(3,4,5)P_3_ (Tanaka-Takiguchi et al., 2009). Depletion of PI(4,5)P_2_ and PI(3,4,5)P_3_ *in vivo* disrupts septin filaments in 3T3 cells (Gilden and Krummel, 2010).

Myosin I is a monomeric, actin-based motor protein with ATPase activity that has been shown to function in the membrane–cytoskeletal interactions, including vesicle transport along actin filaments and regulation of plasma membrane tension. Myosin I molecules have a tail homology (TH) domain that contains a putative phospholipid-binding PH domain. Previous studies have shown that the TH domain preferentially binds to acidic phospholipids such as phosphatidylserine and PI(3,4,5)P_2_. These phospholipids are relatively abundant in biological membranes and their concentrations do not appear to change a great deal in response to intracellular signaling. In contrast, PI(3,4,5)P_3_ levels are highly regulated and function as signaling mechanisms for myosin (Chen et al., 2012).

#### Microglial Chemotaxis and Migration Within the Neurodegenerative Disease

Many neurodegenerative conditions present with alterations in microglial migration and distribution. Microglia follow gradients of chemokines towards damaged and dying cells, which by definition are present in these disorders.

The net migration of microglia induced by deposits of Aβ in AD is well documented. This process acts to concentrate microglia around Aβ deposits in an attempt to neutralize or prevent further damage. Increased levels of a wide range of chemokines have been reported in AD patients (Koenigsknecht-Talboo and Landreth, 2005). One example is MCP-1. Levels of MCP-1 within CSF increase throughout AD and levels correlate with disease severity (Galimberti et al., 2006). Other chemokines including IL-18, VEGF, and Fractalkine have also been shown to be elevated in patients with AD (Kalaria et al., 1998; D’Andrea et al., 2004; Franciosi et al., 2005). In PD α-synuclein aggregates released from neurons activate microglia and act as chemoattractants that direct microglial migration by acting on NADPH oxidase and several other specific downstream proteins (Wang et al., 2015). In HD mutant mHTT protein has been shown to impair immune cell migration by disrupting actin remodeling (Kwan et al., 2012). Notably, PI3K signaling, *via* inhibition of the Akt/Erk signaling cascade, has been shown to significantly contribute to the pathogenesis of AD, PD, and HD (Rai et al., 2019). Within ALS, microglia appear to be less mobile than controls in cellular models when using MCP-1 as a chemoattractant (Yamasaki et al., 2010).

#### Targeting Microglial Chemotaxis and Migration to Treat Neurodegenerative Disease

Targeting chemotaxis to treat neurodegeneration represents a very nuanced problem. While microglia do have a protective role in many forms of neurodegenerative disease, they also have a detrimental role. While an increased number of microglia may be able to reduce damage and clear extracellular proteins they can also initiate a large, potentially damaging neuroinflammatory response. As such promoting chemotaxis to encourage microglia response to damage may be counterproductive while at the same time reducing the neuroimmune response is also unadvised. A few therapies linked to chemotaxis have been investigated, however. TREM2 is a receptor upstream of PLCγ2 in microglia. Sequence variations in TREM2 have been demonstrated to increase the risk of AD (Sims et al., 2017, 2020). TREM2 is currently being investigated as a target for AD therapies (Long et al., 2019) and dysregulation of TREM2 has been shown to reduce chemotaxis (Mazaheri et al., 2017). TGFbeta has been shown to downregulate microglia chemotaxis (Huang et al., 2010) and has been investigated as a treatment of AD (Chao et al., 1994). Given the key roles of PIPs in chemotaxis, perhaps co-manipulation of these lipids could enhance therapeutic effects.

## Conclusion

The microglial function is severely impaired in a variety of ways within neurodegenerative conditions, with these cells typically showing heightened activation states from early stages of disease development, often before symptom onset. Alongside disturbances in microglial homeostasis, disruptions in phosphoinositide levels and metabolism are also seen in many of these same conditions. These PIPs appear to play important roles in the regulation of numerous key microglial functions. Together, these observations suggest that the observed microglial dysfunction may arise in part as a result of this lipid dyshomeostasis.

Further research into both the role of microglia and PIP dyshomeostasis within neurodegenerative disease could provide us with much-needed therapeutics for treating these presently incurable conditions. It may be that co-manipulating microglial functions alongside PIP levels could allow us to boost the effectiveness of targeted therapeutics, thus bringing us closer to the ultimate goal of a world without dementia.

## Author Contributions

TP and EM contributed equally to the researching and drafting of the work presented here. All authors contributed to the article and approved the submitted version.

## Conflict of Interest

The authors declare that the research was conducted in the absence of any commercial or financial relationships that could be construed as a potential conflict of interest.
